# His bundle pacing, learning curve, procedure characteristics, safety, and feasibility: Insights from a large international observational study

**DOI:** 10.1111/jce.14064

**Published:** 2019-08-02

**Authors:** Daniel Keene, Ahran D Arnold, Marek Jastrzębski, Haran Burri, Steven Zweibel, Eric Crespo, Badrinathan Chandrasekaran, Sukhbinder Bassi, Nader Joghetaei, Matthew Swift, Pawel Moskal, Darrel P Francis, Paul Foley, Matthew J Shun‐Shin, Zachary I Whinnett

**Affiliations:** ^1^ National Heart and Lung Institute Imperial College London London; ^2^ First Department of Cardiology, Interventional Electrocardiology and Hypertension Jagiellonian University Collegium Medicum Krakow Poland; ^3^ Department of Cardiology University Hospital, Cardiology Geneva Switzerland; ^4^ Department of Interventional Electrophysiology, Hartford Hospital Interventional Electrophysiology Hartford; ^5^ Cardiology Department Great Western Hospitals NHS Foundation Trust Swindon; ^6^ Cardiology Department Sherwood Forest Hospitals NHS Foundation Trust Sutton, Ashfield; ^7^ Department of Cardiology Klinikum Landkreis Erding, Cardiology Munich Germany

**Keywords:** His bundle pacing, His bundle pacing characteristics, His bundle pacing feasibility, His bundle pacing learning curve, physiological pacing

## Abstract

**Background:**

His‐bundle pacing (HBP) provides physiological ventricular activation. Observational studies have demonstrated the techniques’ feasibility; however, data have come from a limited number of centers.

**Objectives:**

We set out to explore the contemporary global practice in HBP focusing on the learning curve, procedural characteristics, and outcomes.

**Methods:**

This is a retrospective, multicenter observational study of patients undergoing attempted HBP at seven centers. Pacing indication, fluoroscopy time, HBP thresholds, and lead reintervention and deactivation rates were recorded. Where centers had systematically recorded implant success rates from the outset, these were collated.

**Results:**

A total of 529 patients underwent attempted HBP during the study period (2014‐19) with a mean follow‐up of 217 ± 303 days. Most implants were for bradycardia indications.

In the three centers with the systematic collation of all attempts, the overall implant success rate was 81%, which improved to 87% after completion of 40 cases.

All seven centers reported data on successful implants. The mean fluoroscopy time was 11.7 ± 12.0 minutes, the His‐bundle capture threshold at implant was 1.4 ± 0.9 V at 0.8 ± 0.3 ms, and it was 1.3 ± 1.2 V at 0.9 ± 0.2 ms at last device check.

HBP lead reintervention or deactivation (for lead displacement or rise in threshold) occurred in 7.5% of successful implants.

There was evidence of a learning curve: fluoroscopy time and HBP capture threshold reduced with greater experience, plateauing after approximately 30‐50 cases.

**Conclusion:**

We found that it is feasible to establish a successful HBP program, using the currently available implantation tools. For physicians who are experienced at pacemaker implantation, the steepest part of the learning curve appears to be over the first 30‐50 cases.

## 1 INTRODUCTION

Since the first report of successful permanent His bundle pacing (HBP) in 2000 by Deshmukh et al,^1^ HBP has emerged as an alternative to right ventricular endocardial pacing for patients with a bradycardia indication for pacing. HBP produces rapid and physiological ventricular activation via the His‐Purkinje conduction system, rather than the nonphysiological activation, which occurs with right ventricular endocardial pacing due to slow cell‐to‐cell conduction of the electrical wavefront. HBP produces a narrow QRS duration and, by preserving physiological ventricular activation, may prevent the development of right ventricular pacing‐induced cardiomyopathy in patients with a bradycardia indication for pacing.^2^ Furthermore, HBP can often correct bundle branch block, resulting in a reduction in QRS duration and improvement in ventricular activation time.^3^

The HBP pioneers encountered several challenges during the early years of HBP, such as low procedural success rates and high and rising thresholds.^1^ However, with the arrival of specialized delivery equipment and a more suitable pacing lead, the published HBP success rates appear to have improved.^4^, ^5^, ^6^ This published experience includes relatively small numbers of patients from a limited number of expert centers. As a result, there have been concerns about whether, with current technology, it is feasible for HBP to be adopted more widely.

In this study, we report the experience of seven international centers who have adopted HBP, with a particular focus on the learning curve for performing HBP, acute implant success rates as well as acute and longer‐term HBP thresholds.

## 2 METHODS

### 2.1 Study design

This is a retrospective, multi‐center observational study designed to evaluate the contemporary real‐world practice of permanent HBP. We focus on indications for pacing and patient characteristics, procedural characteristics, and device follow‐up. The study population included all patients undergoing attempted permanent HBP, for any indication, at seven implanting centers (1 Germany, 1 Poland, 1 Switzerland, 3 United Kingdom, and 1 United States). Baseline patient demographics together with relevant clinical information (QRS duration, NYHA class, LV function, presence of IHD) were recorded. Patients identified as being recruited into blinded randomized controlled trials, including the HOPE‐HF double‐blind randomized crossover trial,^7^ were excluded from this analysis.

### 2.2 Procedural details

Procedural characteristics (indication, presence of pre‐existing device, procedural success, lead/delivery equipment, lead‐generator configuration, fluoroscopy time, HBP thresholds, and programmed stimulation energy) were recorded.

Clinical indications for HBP were classified into the following five categories:

1. Intermittent/permanent high‐degree atrioventricular block (HD‐AVB, including Mobitz type II second‐degree atrioventricular block and complete heart block),
2. Before atrioventricular nodal ablation (AVNA) for management of atrial fibrillation,
3. Sinus node dysfunction (SND, including tachy‐brady syndrome),
4. Slowly conducted atrial fibrillation (AF)
5. Cardiac resynchronization therapy.

Where patients were noted to have multiple indications, only one was recorded—this was the one with the strongest pacing indication.

The pacing responses observed were recorded as selective His bundle capture, nonselective His bundle capture, and myocardial‐only capture as defined using previously described standard criteria.^4^ The criteria used by the different operators whether to accept a His lead position included (a) the His capture threshold, (b) the paced QRS duration (particularly of relevance if intrinsic QRS is broad), and (c) R wave amplitude, (if the His lead is required for sensing). One center also imposed a time limit of 30 minutes.

Pulse width for HBP threshold testing was determined at the individual implanting centers.

The HBP lead delivery method was divided into three categories: the typically used approaches of the fixed curve C315 His sheath (Medtronic, MN) or the deflectable C304 delivery sheaths (Medtronic), and a third category allowing for hybrid approaches using different delivery sheaths. The type of lead implanted to deliver HBP was also recorded. Fluoroscopy time was stratified by both the number of leads implanted and the reported rhythm during implant.

### 2.3 Clinical outcomes

Clinical outcomes (QRS duration, HBP thresholds, and programmed stimulation energy) were collected. These outcomes were reported at implant and at the latest recorded follow‐up. HBP QRS duration was stratified by selective or nonselective capture. Nonselective capture of the His bundle results in the capture of both the local myocardium and the His bundle. The capture of the local myocardium with nonselective pacing produces a pseudo‐delta‐wave on the ECG, which prolongs the surface QRS duration. Programmed stimulation energy was analyzed as voltage and as a function of voltage and pulse width (*V* ^2^ *t*, V = voltage, t = pulse width).

### 2.4 Statistical analysis

Descriptive statistics, including mean and standard deviation, were used, and for within‐patient changes in parameters, paired *t* tests were performed. Differences between groups were assessed using analysis of variance. To assess the impact of experience on pacing parameters, regression models were constructed (ordinary leased squares or proportional odds as appropriate) with a restricted cubic spline with three knots for experience (measured as a number of cases performed by that center). Data analysis was performed using R version 3.3.

## 3 RESULTS

His bundle pacing was attempted in a total of 529 patients, at the seven implanting centers during the study period (2014‐18); permanent HBP was successfully achieved in 466 of these patients. The number of attempted implants per year is shown in the Supporting Information appendix. The baseline demographics are displayed in Table 1. The mean follow up duration was 217 ± 303 days (Range: 2–1447 days, Median 124 days). Of the total patients, 52% were reported to have impaired ventricular function at the time of device implantation.

**Table 1:** Baseline demographics

Age, y	75 ± 11
Male	363 (69%)
NYHA functional class	1.9 ± 0.9 (n = 481)
I	184 (38.3%)
II	157 (32.6%)
III	127 (26.4%)
IV	13 (2.7%)
Left ventricular function	n = 517
Normal (EF > 55%)	248 (48.0%)
Mildly impaired (EF 45‐54%)	72 (13.9%)
Moderately impaired (EF 36‐44%)	91 (17.6%)
Severely impaired (EF < 35%)	106 (20.5%)
12‐lead QRS duration, ms	118 ± 33 (n = 513)
QRS > 120 ms	n = 211
LBBB	107 (50.7%)
RBBB	53 (25.1%)
Nonspecific IVCD	51 (24.2%)
Ischemic heart disease	145 (33.9%, n = 428)
Pacing indication	
Intermittent/persistent high degree AV block	49.5%
Slowly conducted atrial fibrillation	27.8%
Cardiac resynchronization	7.2%
Sinus node dysfunction	8.9%
Pre‐AV node ablation	6.6%

*Note*: Values are mean ± SD or n (%) for all patients in whom permanent HBP was attempted. Where data are missing, n is provided for the patients in whom data are available.

Abbreviations: HBP, His‐bundle pacing; IVCD, interventricular conduction delay; NYHA, New York Heart Association; SD, standard deviation.

### 3.1 Procedural characteristics

#### 3.1.1 Clinical indications

The majority of HBP attempts were performed in patients with intermittent or persistent high‐degree atrioventricular block and 86.2% of procedures were de‐novo device implantations; the remainder were upgrades of previously implanted devices.

#### 3.1.2 Procedural success rates

Overall, successful permanent HBP was achieved in 81% of the 322 patients implanted in the centers, where implant success rates were systematically recorded.

One center with 47 procedures over 3.5 years had a 91% success rate, another with 108 procedures over 1.7 years had an 81% success rate, and the third with 167 procedures over 4.3 years had a 78% success rate.

Patients who had a QRS duration less than 120 ms were more likely to be successfully implanted than patients with a broader QRS (89%, 163/184 vs 71% 98/138; *P* < .0001); the mean QRS duration of all failed implants was 139 ± 33 ms. In patients with broad intrinsic QRS implant, the failure rates were 31.6% for LBBB, 23.1% for RBBB, and 25.0% for nonspecific interventricular conduction delay.

In 39 cases, the specific reason for HBP failure was not recorded. In the 22 cases where this information was available, the reasons for discontinuing the His pacing attempt were as follows: infra‐Hisian block distal to the pacing site at high rates (n = 2), unmappable His signal with unsatisfactory pace‐mapping (n = 8), lead instability/displacement (n = 3), failure to correct bundle branch block (n = 7), and His pacing threshold prohibitively high (n = 2). With experience, successful implantation rates were higher at 87% after 40 implant attempts.

#### 3.1.3 Devices, leads, and delivery equipment

In all patients, the SelectSecure 3830 69 cm lead was used to attempt HBP. In 89.0% of successful HBP cases, the C315 fixed curve sheath successfully delivered the lead. In 10.1% of implants, the deflectable C304 sheath was used and in 0.9%, a C315 sheath was passed through a modified coronary sinus catheter or a coronary sinus guide catheter alone was used to position the His lead.

A single‐lead pacemaker with only a His bundle lead was implanted in 65 patients (14% of successful HBP implants). A dual‐lead pacemaker was implanted in 182 patients (39%). In 36 of these patients, the His bundle lead was connected to the atrial port with a ventricular “back‐up” lead connected to the ventricular port. In 146 patients, the His bundle lead was connected to the ventricular port with a right atrial lead connected to the atrial port; in two such patients, a right ventricular sensing lead was also implanted and connected to the ventricular port along with a His bundle lead via a Y connector.

A total of 219 (47%) patients received triple lead devices. In 152 patients, a CRT‐P device was used, and 67 patients received a CRT‐D device. In almost all CRT devices, an atrial lead was connected to the atrial port, and the His lead and RV (“back‐up”) lead were connected to the ventricular ports; however, alternative configurations were used in a small minority of cases.

**Figure 1:**
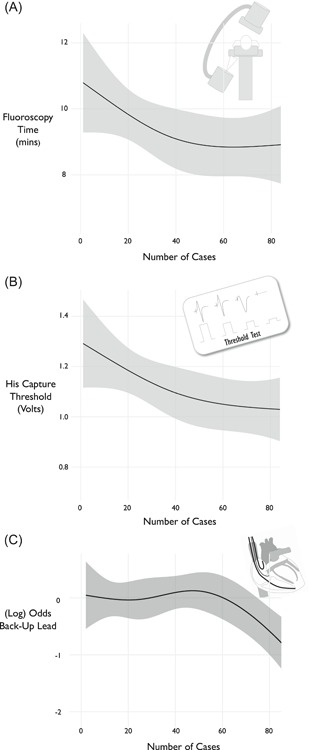
Learning curve metrics A, shows the relationship between fluoroscopy time and center experience (*P* = .15). B, shows the relationship between pacing threshold and center experience (*P* = .04). C, shows the log odds of receiving a back‐up pacing lead in the ventricle (*P* = .0018). Overall successful implantation rate was 81% which increased to 87% after 40 cases. As number of cases per center increased, fluoroscopy time and His capture threshold decreased until plateauing after 30 and 50 cases

From the 399 patients who did not require a mandatory RV lead for defibrillation purposes, 47% were implanted with a “back‐up” ventricular pacing lead in addition to the His lead. Back up lead utilization decreased with experience (*P* = .0018): this is shown in Figure 1.

#### 3.1.4 Fluoroscopy time

The mean fluoroscopy time across all procedures was 11.7 ± 12.0 minutes. Fluoroscopy times, stratified by the number of leads and procedural rhythm, are depicted in Table 2. Overall, fluoroscopy times were not significantly affected by the underlying rhythm during the implant procedure.

**Table 2:** Fluoroscopy times stratified by procedural rhythm and number of leads implanted

Device	Procedural rhythm			
	AF	AVB	SR	All
Single‐lead	10.4 ± 12.0	n/a	n/a	10.4 ± 12.0
	(n = 63)	(n = 0)	(n = 0)	(n = 63)
Dual‐lead^a^	9.7 ± 7.5	9.2 ± 6.3	8.8 ± 7.1	9.2 ± 7.0
	(n = 52)	(n = 55)	(n = 68)	(n = 175)
Triple‐lead^b^	13.1 ± 17.2	11.2 ± 13.6	13.8 ± 13.0	12.8 ± 14.4
	(n = 50)	(n = 59)	(n = 75)	(n = 184)
All	11.0 ± 12.9	10.2 ± 10.7	11.4 ± 10.8	11.7 ± 12.0
	(n = 165)	(n = 114)	(n = 143)	(n = 422)

*Note*: Values are fluoroscopy time (minutes) mean ± SD (number of patients with available data). Two patients data missing from single‐lead device, three patients data missing from dual‐lead device, and nine patients data missing from triple‐lead devices.

Abbreviations: AF, atrial fibrillation; AVB, atrioventricular block; LV, left ventricle; SD, standard deviation; SR, sinus rhythm.

^a^ Excludes four patients implanted with an LV lead.

^b^ Excludes 26 patients implanted with an LV lead.

Thirty implants involved a coronary sinus (CS) lead and were excluded from fluoroscopy time analysis (Table 2) as variation in fluoroscopy use for CS lead implantation is not relevant to this analysis and could be responsible for differences in th overall fluoroscopy time. Fluoroscopy times tended to reduce with increasing His bundle pacing experience (*P* = .15) as shown in Figure 1 with a leveling off after 30‐50 cases.

### 3.2 Clinical outcomes

#### 3.2.1 Initial thresholds and programmed pacing outputs at the implant

The mean threshold of His bundle capture (whether selective or nonselective) was 1.4 ± 0.9 V at 0.8 ± 0.3 ms (data available in 449 cases [96%]). Selective His bundle capture was achieved in 200 patients, with a mean threshold of His bundle capture of 1.4 ± 0.8 V at 0.7 ± 0.3 ms. In 63 of these patients, nonselective capture occurred at pacing outputs above the threshold of selective capture; in these cases, the mean threshold of nonselective capture was 2.6 ± 1.6 V at 0.7 ± 0.3 ms and the mean threshold of selective capture was 1.1 ± 0.5 V at 0.7 ± 0.3 ms.

In the remaining 249 patients, selective His bundle capture was not observed, the mean threshold of His bundle capture in this group was 1.4 ± 1.0 V at 0.9 ± 0.3 ms. In 64 of these patients, the myocardial capture threshold was reported and found to be lower than the nonselective threshold with a mean myocardial threshold of 0.9 ± 0.6 V at 0.9 ± 0.2 ms.

**Figure 2:**
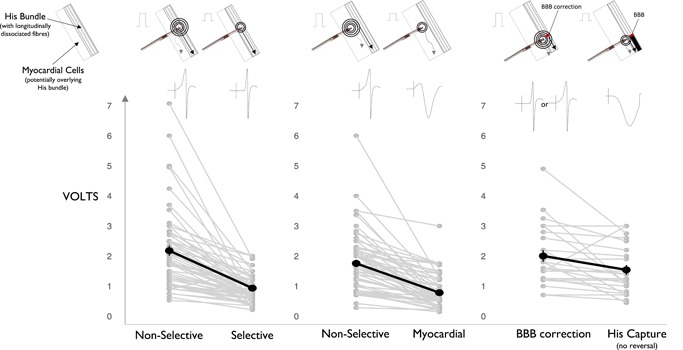
Within patient change in thresholds for different pacing responses seen with His bundle pacing. Cartoon illustrations are shown above depicting what may be occurring with a change in voltage amplitude

In patients with bundle branch block where either the left or right bundle was corrected by His bundle pacing (n = 27), the mean threshold for bundle recruitment was 2.1 ± 1.0 V at 0.9 ± 0.2 ms and the mean threshold of His bundle capture (without necessarily recruiting both bundles) was 1.6 ± 0.8 V at 0.9 ± 0.2 ms. Figure 2 depicts the within‐patient change in thresholds for the different pacing responses seen.

The relationship between the threshold of His bundle capture and center experience is shown in Figure 1. As experience increased, the threshold of His bundle capture reduced, before plateauing (*P* = .04), again demonstrating a learning curve.

The mean programmed His lead pacing output across all cases at implant was 4.2 ± 1 at 0.9 ± 0.2 ms (data available in 322 cases) with a mean safety margin of 2.9 ± 1.2 V above the threshold of His bundle capture.

#### 3.2.2 Last available follow‐up thresholds and programmed stimulation outputs

The mean threshold of His bundle capture (whether selective or nonselective) at the last available follow‐up was 1.3 ± 1.2 V at 0.9 ± 0.2 ms (n = 366). Mean programmed His lead pacing output at follow‐up was 3.0 ± 1.1 V at 0.8 ± 0.3 ms with a safety margin of 1.7 ± 1.0 V above the threshold of His bundle capture. The details of all thresholds for the various types of His bundle pacing are recorded in Table 3.

**Table 3:** His pacing thresholds

	Initial threshold	Follow up threshold
Lowest threshold of His bundle capture (Selective or nonselective)		
** **All HBP	1.4 ± 0.9 V	1.3 ± 1.2 V
	0.8 ± 0.3 ms	0.9 ± 0.2 ms
	(N = 449)	(N = 366)
His bundle captured selectively only		
** **Selective HBP	1.4 ± 0.8 V	1.1 ± 1.0 V
	0.7 ± 0.3 ms	0.9 ± 0.2 ms
	(N = 200)	(N = 152)
Nonselective HBP threshold above selective HBP threshold		
Nonselective HBP	2.6 ± 1.6 V	2.4 ± 1.7 V
	0.7 ± 0.3 ms	1.0 ± 0.1 ms
	(N = 63)	(N = 82)
Selective‐HBP	1.1 ± 0.5 V	0.8 ± 0.5 V
	0.7 ± 0.3 ms	0.9 ± 0.2 ms
	(N = 63)	(N = 82)
His bundle is only captured nonselectively		
** **Nonselective HBP	1.4 ± 1.0 V	1.5 ± 1.7 V
	0.9 ± 0.3 ms	0.9 ± 0.1 ms
	(N = 249)	(N = 214)
** **Pure myocardial capture	0.9 ± 0.6 V	1.0 ± 0.8 V
	0.9 ± 0.2 ms	1.0 ± 0.2 ms
	(N = 64)	(N = 59)
Intrinsic BBB corrected by HBP		
** **BBB correction	2.1 ± 1.0 V	2.1 ± 1.1 V
	0.9 ± 0.2 ms	0.9 ± 0.3 ms
	(N = 27)	(N = 21)
** **Lowest HBP	1.6 ± 0.8 V	1.3 ± 1.0 V
	0.9 ± 0.2 ms	0.9 ± 0.3 ms
	(N = 27)	(N = 21)

*Note*: Values are mean threshold (V) ± SD at mean pulse width (ms) ± SD. N is the number of patients with available data.

Abbreviations: HBP, His‐bundle pacing; SD, standard deviation.

Of the 366 patients with follow up threshold data available, 31 (8.5%) had a rise in threshold to 3 V or more. Of these patients, the mean threshold at implant was 2.01 V ± 1.1 with a rise to 4.3 V ± 1.5 during follow‐up. Not all of these changes required reintervention, for example in three patients (with small rises <0.5 V) the patients had previously undergone failed LV lead implantation and the (“bail‐out”) His pacing system was delivering satisfactory ventricular resynchronization.

HBP threshold data were not available for all patients at follow‐up (100 patients). The reasons for this include: initial implant within 6 weeks and first follow‐up not occurred (n = 58), data not reported (although no mention of lead‐related issues included in supplied data set) (n = 17), patient death (n = 5 [3 progressive heart failure, 2 unknown]), patient followed up at different hospital (n = 1), pacemaker explanted due to infection (n = 1) or explanted due to venous thrombosis (n = 1).

In 17 patients, no follow up capture threshold data was reported because they were not receiving His pacing. In three patients, this was because there was only myocardial capture present (n = 3) (mean threshold 0.5 V at 1ms). In 14 patients, His lead had been deactivated. Reasons for deactivation were a rise in threshold (n = 6) and displacement and loss of capture (n = 7). One patient experienced exercise‐induced symptoms and 2:1 infra‐Hisian block was observed during an exercise test. Therefore, His lead was deactivated and RV only pacing was performed.

#### 3.2.3 His bundle lead complications

In addition to the 17 patients without follow‐up threshold data reported due to His lead issues, eight additional patients had undergone lead revisions either due to displacement (n = 5) or unsatisfactory threshold rise (n = 3). In further 10 patients, the His pacing lead had been deactivated due to displacement and loss of capture (n = 3) or due to unsatisfactory threshold rise (n = 7). Therefore, overall 7.5% of initially successful His lead implants experienced issues requiring either a lead revision or lead deactivation.

#### 3.2.4 ECG responses

Mean preimplant QRS duration across all attempted patients was 118 ± 33 and 116 ± 31 ms in those successfully implanted. Across the group as a whole, there was no significant difference in paced QRS duration, which was 115 ± 24 ms (*P* = .5).

In patients with an intrinsic QRS duration <120 ms, the mean within‐patient change from intrinsic QRS to selective His bundle capture QRS was +8 ± 18ms (n = 125) (*P* < .0001) and the mean within‐patient change from intrinsic QRS to nonselective His bundle capture QRS was +17 ± 23 ms (n = 130) reflecting the presence of a pseudo‐delta‐wave on the ECG (*P* < .0001).

**Figure 3:**
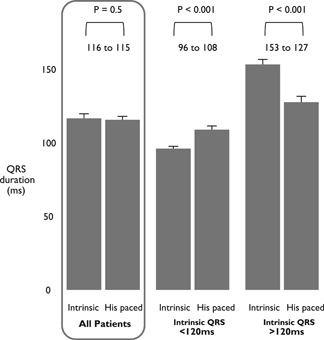
Summary of QRS duration changes for all patients receiving His bundle pacing and then stratified according to intrinsic QRS duration either less than or more than 120 ms. (Error bars are standard error of the mean)

In patients with intrinsic QRSd > 120 ms, the mean within‐patient change in QRS duration from intrinsic QRS to selective His bundle capture QRS was −21 ± 29 ms (n = 60) and the mean within‐patient change in QRS duration from intrinsic QRS to nonselective His bundle capture QRS was −27 ± 32 ms (n = 102) (*P* < .0001) (this narrowing was despite the presence of a pseudo‐delta wave). Figure 3 shows the summary of ECG changes, and full details are provided in Table 4.

**Table 4:** Effect of His bundle pacing on QRS duration

Group	QRS duration (ms)
All attempted implants preprocedure (n = 513)	118 ± 33
All successful implants stratified by His Pacing only (n = 417)	
Presuccessful implant	116 ± 31.6
Post successful implant	115 ± 23.8
All successful implants stratified by selective or nonselective capture	
Selective capture (n = 185)	Pre 113 ± 28.5
	Post 113 ± 22.4
Nonselective capture (n = 232)	Pre 118 ± 33.9
	Post 117 ± 24.6
All successful implants stratified by intrinsic QRS duration and by selective or nonselective capture	
Preimplant QRS < 120 ms	Pre 97 ± 11.7
Selective capture reported	Post 105 ± 18.4
(n = 125)	Diff 7.6 ± 17.5
Preimplant QRS < 120 ms	Pre 93 ± 12.2
Nonselective capture reported	Post 111 ± 20.9
(n = 130)	Diff 17.0 ± 23.0
Preimplant QRS > 120 ms	Pre 149 ± 21
Selective capture reported	Post 127 ± 20.6
(n = 60)	Diff –21.3 ± 29.0
Preimplant QRS > 120 ms	Pre 152 ± 22.4
Nonselective capture reported	Post 126 ± 26.7
(n = 102)	Diff –27. ± 32

## 4 DISCUSSION

This is a large cohort of His bundle pacing and covers a period of adoption in seven international centers. Implant success rates were satisfactory overall. There was clear evidence of a learning curve with progressive accumulation of experience, there was progressive reduction in (a) fluoroscopy time, (b) His bundle capture thresholds, and (c) reliance on back up leads. This learning curve appears to occur in over 30 to 50 cases. Neither acute nor chronic His pacing thresholds were excessively high. His leads were found to require reintervention or deactivation in 7.5% of cases.

### 4.1 Implant success rates

The overall success rate of attempted HBP was 81%. After the first 40 implants at a center, the success rate was higher at 87%. Other present‐day reports of HBP success rates are consistent with this, ranging from 80% to 92%.^4^, ^8^, ^9^ Some hospitals did not have comprehensive success rate data and it is conceivable that their rates may have been lower. Nevertheless, these rates compare favorably to the pioneering reports of HBP, which did not benefit from specialized delivery systems, having success rates as low as 66%.^1^

The current 87% success rates that can be expected by patients undergoing implantation at experienced centers are the result of both improvements in lead delivery approach and also the growth of experience by operators at the implanting centers. Further increases in collective operator experience and advancements in delivery tools may bring HBP success rates up to the level now seen in biventricular pacing. In fact, since biventricular pacing was the previous such pacing revolution, it is notable that after a decade of experience major RCTs were reporting success rates (83% in PAVE 10 and 82% in RD‐CHF^11^) similar to the HBP success rates in this study. Importantly, HBP leads and delivery equipment can also be used for conventional atrial or ventricular pacing and even distal conduction system pacing where the left bundle itself is directly stimulated, offering flexibility when HBP capture cannot be achieved.^12^

### 4.2 His pacing safety

We found that 7.5% of His pacing leads required either a repeat intervention or were deactivated. The most common reasons for this were lead displacement and rise in pacing threshold. This is similar to the 6.7% rate reported in a prior long term follow‐up study of His bundle pacing^5^ and is comparable to the approximately 7% LV lead revision rate seen in 15 222 patients included in the studies of biventricular pacing.^13^ Indeed, given the decades more of technical developments for RV leads, it is remarkable that the rate of RV lead repeat interventions is still even now as high as 3%.^14^ While these rates for His pacing are not yet down to the level of RV pacing, they are close enough for a trial to be warranted to determine whether the physiological advantages already outweigh the disadvantages of lead revisions.

### 4.3 Choice of lead and delivery kit

The SelectSecure 3830 lead (Medtronic) has emerged as the current dominant lead used for His bundle pacing and was used in every case in this study. Its popularity arises from its design of having the screw tip itself as the active electrode so that the pacing can be precisely delivered, even to a His bundle site embedded deep within the myocardium. If other leads, which do not have this feature, are used, the cathode pacing electrode is separate from the screw which means the electrical impulse potentially needs to pass through several millimeters of tissue between the electrode and the His bundle. The other reason for the popularity of this lead is that dedicated delivery sheaths have been developed to deliver it to the His bundle.

The C315His fixed curve delivery sheath appears to be sufficient in most cases to facilitate HBP, with a different sheath required in only 11% of cases. The shape of the C315 sheath is not ideal for all patients, particularly in cases where the right atrium is dilated or when there is ventricular dilatation and therefore displacement of the tricuspid valve and His bundle region. In our study, the deflectable C304 sheath was most often used when the C315 sheath failed to deliver the lead to the His bundle region. However, this was also not optimal in all cases and we found that operators improvised with alternative solutions. For example, modified coronary sinus sheaths were used or combined with the C315 sheath so that extra reach could be achieved while maintaining perpendicular delivery of the lead to the septum. New lead delivery systems are presently in the testing phase and when available, they will provide operators more options, which will hopefully further increase the success rate of His bundle pacing.

### 4.4 Back‐up pacing leads

When a defibrillator lead is not indicated in a patient receiving a His lead, operators may decide to implant a backup pacing lead, typically in the right ventricle. The potential reasons for considering implanting a backup pacing lead are as follows: First, it provides a back‐up option of pacing in case of loss capture from the His lead. Loss of capture may occur due to lead displacement, fibrosis at the lead tip or the development of progressive conduction system disease distal to the site of lead implantation. Second, it may be used for sensing in cases where the R wave amplitude recorded from the His lead is small and/or the atrial or His potentials are large, which increases the risk of R wave under‐sensing or atrial/His oversensing (potentially leading to asystole in a pacing‐dependant patient). Third, if there is a high His capture threshold, a lower safety margin may be considered to preserve generator longevity, if an RV back up pacing lead is available. Fourth, in case of selective HBP with uncorrected RBBB, a fusion between His and RV pacing can provide narrowing of the QRS, which has recently been shown to be of clinical interest.^15^

However, there are also potential disadvantages of using a backup lead. First, implanting more leads is associated with an increased risk of infection.^16^ Second, placing an RV lead, particularly at the RV apex, has a small but potentially catastrophic risk of causing cardiac perforation. Third, RV leads may interact with the tricuspid valve to cause tricuspid valve regurgitation. Fourth, an increased number of leads may predispose to venous stenosis and a higher risk of complications, if lead extraction is required. Fifth, in the absence of dedicated His pacing algorithms, the back‐up lead is often programmed to pace after His bundle lead, which increases battery drain compared to pacing from a single lead.

The most likely reason for the observed reduction of the use of backup pacing in our study is that with increasing experience of pacing follow up of His leads that operators develop greater confidence in the stability of leads positioned in the His bundle position as well as the reliability of His bundle pacing. An appreciation that back‐up pacing may be provided through nonselective capture, where both the His bundle itself and the surrounding myocardium are activated by the pacing stimulus, also provides reassurance to operators. With nonselective capture, myocardial activation typically occurs even if His bundle capture fails or if there is the development of distal conduction system disease. Second, improved sensitivity algorithms now allow even relatively small R waves to be sensed satisfactorily.

Greater operator experience, improvements in delivery systems and lead design, and the development of dedicated His bundle sensing algorithms may lead to further future reductions in the use of back‐up pacing leads.

The present study shows that after approximately 40 cases, there is a clear decline (*P* = .0018) in the rates of back‐up lead implantation (Figure 1).

### 4.5 Fluoroscopy times are low and show a learning curve

Fluoroscopy time is important as a measure of procedural feasibility and is an indirect index of the overall procedure duration. The mean fluoroscopy time for HBP was acceptably low across all patients. A key finding from our study is evidence of a learning curve for HBP. Fluoroscopy times became shorter as experience increased. The decline in fluoroscopy time levelled out after 30 to 50 cases. This is very useful information when centers are planning to start His bundle pacing programs.

### 4.6 Thresholds are low and also show a learning curve

Our study shows that fears of HBP thresholds being high at implant and rising with time are not borne out in contemporary practice. The mean HBP threshold was found to be acceptable at 1.4 V @ 0.8ms. During the follow‐up period, there was no significant deterioration in the capture threshold. Notably, the increasing experience of His bundle pacing was associated with a reduction in implant His pacing thresholds. This process continued until a plateau formed after around 40 cases (Figure 1). This provides reassurance that His bundle pacing is a feasible alternative to RV pacing.

Even though the His bundle is small, it has regions along with it with different pacing parameters. Potential explanations for our finding of improvements in His pacing thresholds with increasing experience may result as with increasing experience, operators become increasingly confident that they will be able to reposition the lead back on the His bundle should they elect to reposition it if the threshold in the first position is higher than desired. With greater experience, operators may also be able to more frequently deliver the lead perpendicular to the septum with better resultant pacing parameters.

### 4.7 ECG capture responses

Patients with QRS > 120 ms showed clear shortening in QRS duration with HBP, with a mean reduction of 26 ms in this study. Patients with QRS < 120 ms showed small but statistically significant increases in QRS duration. This effect was particularly small in those with selective capture.

Even though one may, at first, expect nonselective capture to broaden QRS duration, if the intrinsic QRS is broad, it can be narrowed. Across our patients with nonselective capture, there was essentially no change in the mean QRS with pacing. This arose from a significant QRS widening in those with an intrinsic QRS < 120 ms and a significant QRS narrowing in those with an intrinsic QRS > 120 ms.

### 4.8 Limitations

This is an observational, registry data set reliant on physicians reporting of data not acquired prospectively. Data on R wave sensing were not available from all centers and therefore we were unable to report on sensing in this study. Although most centers were not recruiting into randomized control trials, at least three were: this may have an influence on the data derived for learning curves.

Only three centers systematically collected implant success rate data. It is possible that implant success rates may vary depending on implant volume. Our study did not address this question which will need to be addressed in a prospective study.

There was no ECG core lab for confirming His capture; therefore the presence and type of His bundle capture were verified by the implanting physician according to the accepted criteria published in the His pacing consensus document.^17^ The method for measuring QRS duration was determined by the local protocol within each institution. Although this was standardized within an institution, it was not standardized across the whole study.

As this was a retrospective study, there was not a strict protocol regarding the His pacing threshold and R wave amplitude accepted. Individual operators made this decision on clinical grounds.

## 5 CONCLUSION

This large, contemporary, and global experience of HBP shows that current practice in HBP has acceptable procedural success rates, acceptably low fluoroscopy time, low and overall reliable His bundle capture thresholds. The current deactivation or reintervention rate is not prohibitive and is similar to that of LV leads.

The learning curve for achieving His bundle pacing appears to plateau after around 40 cases with a progressive reduction in fluoroscopy time and His bundle capture threshold.

## Supporting information

Supporting information
