# Supplementary figures and images for: His bundle pacing, learning curve, procedure characteristics, safety, and feasibility: Insights from a large international observational study
Source: J Cardiovasc Electrophysiol. 2019 Aug 2;30(10):1984–93. doi: 10.1111/jce.14064 (PMC7038224; doi:10.1111/jce.14064)

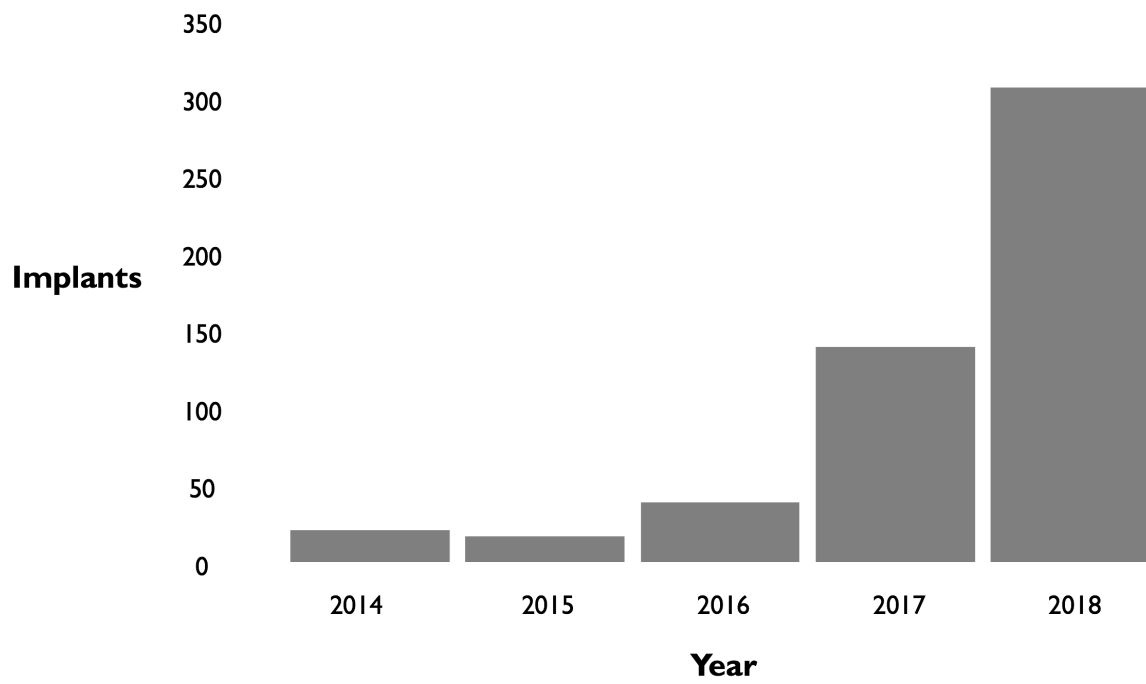

Supplementary Figure – Implants per year over course of the registry.

Supplement: Supplementary file 1 — Supporting information [file JCE-30-1984-s001.pdf]
